# Use of Ferulic Acid in the Management of Diabetes Mellitus and Its Complications

**DOI:** 10.3390/molecules27186010

**Published:** 2022-09-15

**Authors:** Xu Li, Jingxian Wu, Fanxing Xu, Chun Chu, Xiang Li, Xinyi Shi, Wen Zheng, Zhenzhong Wang, Ying Jia, Wei Xiao

**Affiliations:** 1 Jiangsu Kanion Pharmaceutical Co., Ltd., Lianyungang 222001, China; 2 Wuya College of Innovation, Shenyang Pharmaceutical University, Shenyang 110016, China; 3 School of Pharmacy, Shenyang Pharmaceutical University, Shenyang 110016, China; 4 School of Pharmaceutical Engineering, Shenyang Pharmaceutical University, Shenyang 110016, China; 5 Faculty of Functional Food and Wine, Shenyang Pharmaceutical University, Shenyang 110016, China

**Keywords:** ferulic acid, diabetes mellitus, diabetic complications

## Abstract

Diabetes mellitus, a metabolic disease mainly characterized by hyperglycemia, is becoming a serious social health problem worldwide with growing prevalence. Many natural compounds have been found to be effective in the prevention and treatment of diabetes, with negligible toxic effects. Ferulic acid (FA), a phenolic compound commonly found in medicinal herbs and the daily diet, was proved to have several pharmacological effects such as antihyperglycemic, antihyperlipidemic and antioxidant actions, which are beneficial to the management of diabetes and its complications. Data from PubMed, EM-BASE, Web of Science and CNKI were searched with the keywords ferulic acid and diabetes mellitus. Finally, 28 articles were identified after literature screening, and the research progress of FA for the management of DM and its complications was summarized in the review, in order to provide references for further research and medical applications of FA.

## 1. Introduction

Diabetes mellitus (DM), a chronic noncommunicable disease mainly characterized by hyperglycemia, can cause various life-threatening health problems. The main types of DM include type 1 diabetes mellitus (T1DM), type 2 diabetes mellitus (T2DM), gestational diabetes mellitus (GDM), and other types of DM [1]. According to the latest epidemiological data, as one of the fastest growing diseases globally, the number of adults with DM is estimated to increase to 643 million by 2030, and 783 million by 2045 [2]. Traditional phytomedicines are used worldwide for the treatment of a range of diseases, including diabetes, and the use of natural ingredients for diabetes treatment has become a focus of clinical research [3].

**Figure 1:**
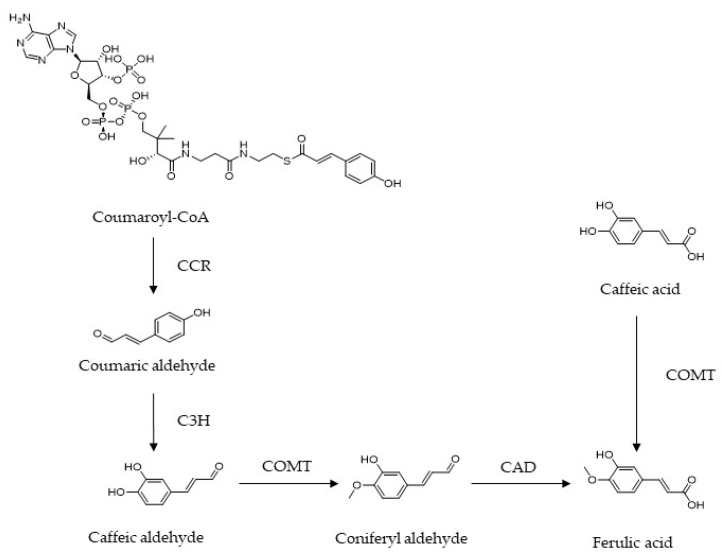
Biosynthesis pathway of ferulic acid. CCR, cinnamoyl-CoA reductase; C3H, p-coumarate 3-hydroxylase; COMT, caffeic acid O-methyltransferase; CAD, coniferyl-aldehyde dehydrogenase.

Ferulic acid (FA) ((E)-3-(4-hydroxy-3-methoxy-phenyl) prop-2-enoic acid), a caffeic acid derivative, not only can be isolated from Chinese herbal medicines including *Cimicifuga racemosa*, *Angelica sinensis*, and *Rhizoma Ligustici Chuanxiong*, but also exists in our daily diet, such as *Oryza sativa*, *Glycine max*, *Zea mays*, *Triticum aestivum *L., and *Avena sativa* Linn. [4]. There are two effective pathways for synthesizing FA in plants. For the phenylpropanoid metabolic pathway, cinnamyl-CoA reductase (CCR) catalyzes the conversion of coumaroyl-CoA to coumaric aldehyde, which is hydroxylated into caffeic aldehyde under the action of coumarin 3-hydroxylase (C3H). As an intermediate of this pathway, caffeic aldehyde can be transformed into coniferyl aldehyde in the presence of caffeic acid O-methyltransferase (COMT), and eventually converted into FA, dependent on conifer aldehyde dehydrogenase (CAD). Alternatively, FA can be obtained by caffeic acid under the catalysis of COMT [5,6,7] (Figure 1).

**Figure 2:**
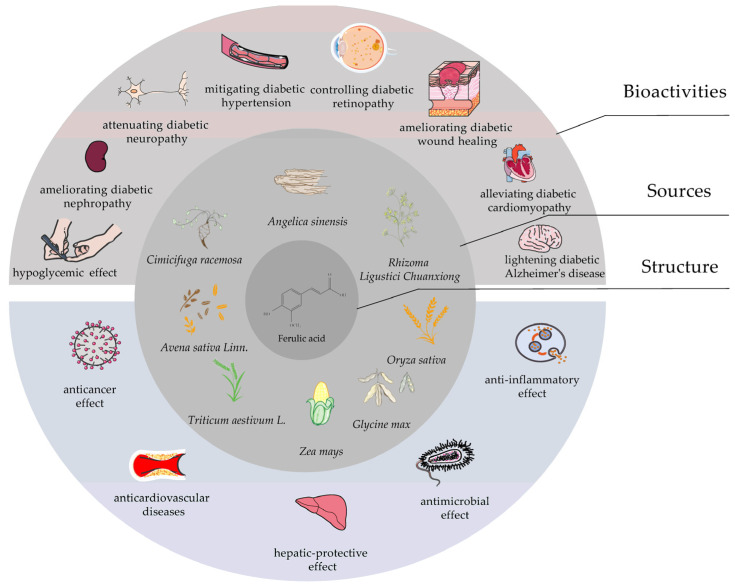
Structure, sources and bioactivities of ferulic acid.

FA possesses free radical scavenging and antioxidant activities which present a wide range of potential effects in the control of cancer and cardiovascular diseases, as well as in hepatic-protective, antimicrobial and anti-inflammatory treatments, especially in the prevention and treatment of DM and its complications [8,9,10,11,12,13,14] (Figure 2). 

**Figure 3:**
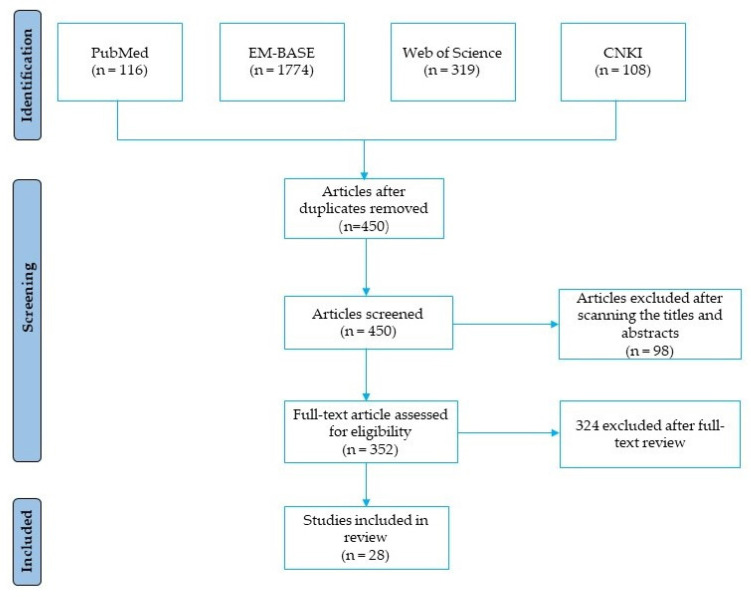
The flowchart of study selection for this systematic review.

Our search strategy is designed according to the characteristics of different databases. PubMed, EM-BASE, Web of Science and CNKI were searched for relevant studies up until July 2022 using the following keyword combination: “Ferulic acid AND Diabetes Mellitus”. In the first stage, articles were searched for using the selected keywords. In the second stage, titles and abstracts of all papers were screened. The third step was to screen selected titles individually in detail to determine whether they were appropriate for the purpose of the study. Our pre-set search strategy returned 116, 1774, 319, and 108 publications from PubMed, EM-BASE, Web of Science, and CNKI, respectively. After browsing the title and abstract or reading the full text, a total of 28 publications were selected in our review (Figure 3).

In this paper, the therapeutic effects of FA on DM and its complications are summarized to provide references for the further development and utilization of FA.

## 2. Use of FA to Prevent and Treat DM

### 2.1. The Effects of FA on Hepatic Glucose Production

The liver is one of the most important organs in the human body, and it plays an indispensable role in regulating glucose and lipid metabolism [15]. During the development of DM, insulin resistance in the liver causes an increase in the level of hepatic glucose production via different pathways, such as inhibiting glycolysis, aerobic oxidation and glycogen synthesis, and promoting the gluconeogenesis [16]. Moreover, DM causes damage to multiple organs, especially the liver. Liver injury caused by DM is mediated, at least partly, by oxidative damage, which can be alleviated by antioxidants [17,18]. With an aromatic phenolic ring, FA can stabilize and delocalize unpaired electrons within it and was used as a free radical scavenger [19]. Balasubashini et al. (2004) found that oral gavage with 10 mg/kg FA for 45 days reduced streptozotocin (STZ)-induced oxidative stress in the liver of Wistar rats by increasing the activities of antioxidant enzymes such as glutathione peroxidase (GPx), superoxide dismutase (SOD) and catalase (CAT) [20]. Consistently, after treating the high-fat and fructose-induced diabetic Wistar rats with intragastrical (i.g.) administration of FA (50 mg/kg) for 30 days, the animals displayed a normal range of blood glucose, serum insulin, glucose tolerance and insulin tolerance with decreased hepatic glucose production in the liver tissue [11]. The hepatic glucokinase (GK) enzyme plays an important role in the regulation of glucose homeostasis, which improves the utilization of blood glucose to generate energy and promotes hepatic glycogen storage. Furthermore, glucose-6-phosphatase (G6Pase) and phosphoenolpyruvate carboxykinase (PEPCK) are the key enzymes that regulate hepatic gluconeogenesis and glucose production [21]. Son et al. (2010) revealed that FA effectively enhanced the level of glycogenesis by increasing the activity of hepatic GK enzyme, and reducing the activities of G6Pase and PEPCK [19]. These results that suggest FA can regulate glucose homeostasis by ameliorating hepatic glucose metabolism disorder.

### 2.2. The Effects of FA on β-Cell Function

The pancreatic β-cell is an endocrine cell type with the unique function of synthesizing, storing and secreting insulin to decrease blood glucose concentration [22]. Pancreatic β-cells are extremely sensitive to oxidative stress [23,24], and their dysfunction may be caused by an imbalance between the production of free radicals and the defense function of antioxidant enzymes [18,25]. Previous studies have shown that hyperglycemia can reduce the activity of antioxidant enzymes in pancreatic islets, and cause the formation of free radicals as a sign of oxidative stress [26]. Oral gavage of FA (10 mg/kg, 45 days) has been proved to effectively neutralize STZ-induced free radicals in the pancreas of diabetic animals induced by STZ, and reduce the toxicity of STZ through its antioxidant properties [4,20,27]. The use of FA (20 mg/kg, i.g. once daily for 12 weeks) was found to inhibit the apoptosis of β-cells in pancreatic islets and protect placental tissue in diabetic gestational Sprague Dawley (SD) rats fed with a high-fat diet in another study [28]. In other related work, chronic hyperglycemia was shown to trigger an oxidative stress response, resulting in increased lipid oxidation in pancreatic tissues, while oral administration of FA (50 mg/kg) for 8 weeks significantly reduced lipid peroxidation in pancreatic tissues to protect STZ-induced β-cell injury in Wistar rats [29]. Importantly, another study suggested that the combination of metformin (12.5 mg/kg) and FA (10 mg/kg) for 3 weeks by oral gavage improved STZ-impaired β-cell regeneration in Wistar rats. From this point of view, the co-administration of FA and metformin could improve the mass of functional β-cells, thus exerting a hypoglycemic effect [13,30]. 

### 2.3. The Effects of FA on Lipid Metabolism

Lipid metabolism is closely related to the development of DM. Insulin secreted from β-cells not only played a role in glucose metabolism, but also mediated lipid metabolism. Insulin resistance can reduce the utilization of glucose in the body, and in obese subjects, excessive lipid makes cells less sensitive to insulin [31]. Over the past few years, many studies have revealed the important role of the phosphoinositide-3 kinase (PI3K)/protein kinase B (Akt) signaling pathway in maintaining insulin sensitivity [32]. Lipogenesis is an insulin- and glucose-dependent process regulated by sterol regulatory element binding protein 1c (SREBP1c), a signaling molecule downstream of Akt, and carbohydrate response element binding protein (ChREBP), ultimately affecting lipid metabolism [33,34]. A previous study showed that oral administration with FA at doses of 25 and 50 mg/kg daily for 8 weeks significantly lowered the insulin resistance and decreased the levels of plasma triglycerides (TG), free fatty acid (FFA), cholesterol and phospholipids in rats fed with a high-fat diet. Additionally, the modulation of FA in lipid homeostasis was further found to be related to the decreased expression of lipogenic genes, such as SREBP1c, fatty acid synthase (FAS), acetyl-CoA carboxylase (ACC), as well as the upregulated expression of β-oxidation genes such as hepatic carnitine palmitoyltransferase 1a (CPT1a) and peroxisome proliferator-activated receptor alpha (PPARα) in liver tissues [35]. An increased level of FFA could also induce the production of acetyl-CoA and cholesterol [36]. As the main site of insulin resistance, excessive lipid accumulation in the liver further leads to reduced glucose uptake and an increased level of blood glucose [19]. Sri Balasubashini et al. (2003) revealed that the levels of hydroperoxides and FFA in the liver of STZ-induced diabetic Wistar rats were reduced after treatment with FA at 10 mg/kg by gavage for 45 days, suggesting that FA improves lipid metabolism with an alleviation of oxidative stress [37]. Accordantly, another in vivo study suggested that feeding STZ-induced diabetic mice with a 0.01% FA-containing diet ad libitum for 7 weeks reduced thiobarbituric acid reactive substances (TBARS) to inhibit the lipid peroxidation in brown adipose tissue [38]. 

The available data on anti-diabetic activities related to FA are shown in Table 1. The dosage, routes of administration, duration of treatment, animal models as well as molecular mechanisms are all listed in detail.

## 3. Use of FA to Prevent and Treat DM Complications

### 3.1. The Effects of FA on Diabetic Nephropathy

Diabetic nephropathy (DN) is one of the most common microvascular complications of diabetes, and has become one of the main causes of death in end-stage renal disease (ESRD) [39]. Many pathways have been confirmed to be involved in the pathogenesis of DN so far, including the mitogen-activated protein kinases (MAPKs)/extracellular regulated protein kinases (Erk1/2) signaling pathway, the PI3K/Akt signaling pathway and the advanced glycation end products (AGEs) pathway [40,41,42]. Currently, there is a growing interest in the use of FA for the treatment of DN. A recent study suggested that FA (100 mg/kg, i.g. once a day for 8 weeks) can markedly improve the renal organ coefficient, increase activities of SOD, CAT, and GPx, and alleviate STZ-induced pathological damage of renal tissue in diabetic rats [39]. Another study revealed that the oral administration of FA at the dose of 50 mg/kg for 8 weeks can significantly ameliorate renal cell apoptosis, inflammation and defective autophagy in diabetic rats by modulating AGEs, nuclear factor kappa-B (NF-κB), MAPKs protein 38 (P38), c-Jun N-terminal kinase (JNK) and Erk1/2 signaling pathways [43]. Oxidative stress leads to increased expression of cyclooxygenase-2(COX-2), intercellular cell adhesion molecule-1 (ICAM-1), and vascular endothelial cells adhesion molecule 1 (VACM-1) in the renal cortex. COX-2 could induce glomerular hyperfiltration, while ICAM-1 as well as VACM-1 are known to induce monocyte invasion [44,45,46,47]. Supplementation with FA at a dose of 10 mg/kg for 20 weeks significantly reduced the thickness of the glomerular basement membrane, the glomerular volume and mesangial matrix expansion in Otsuka Long-Evans Tokushima Fatty (OLETF) diabetic rats’ kidneys. Additionally, further studies revealed that the level of monocyte chemotactic protein 1 (MCP-1) was significantly decreased in cultured podocytes after treatment with FA (10 μM) [48]. Overall, FA may prevent and treat DN, at least partly, by reducing renal oxidative stress.

### 3.2. The Effects of FA on Diabetic Neuropathy

Diabetic neuropathy (DPN) is another common complication of diabetes which affects 30~90% of diabetic patients globally [49]. In a hyperglycemic environment, nerve cells and fibers are prone to pathological changes which can be manifested as impaired vascular function, and lack of angiogenesis and neurotropic factors in nerves [50,51,52]. In addition, multiple signaling pathways including the polyol pathway, hexosamine pathway, AGEs pathway, PARP pathway, MAPK pathway, NF-κB pathway and tumor necrosis factor-α (TNF-α) pathway are involved in the development and pathogenesis of DPN [53]. Previous studies found that the levels of TNF-α, IL-1β and COX-2 were upregulated in SD rats with DM after of 10 IU/kg insulin (s.c.) and 100 mg/kg FA (i.g.) for 4 weeks compared to streptozotocin (STZ)-induced diabetic control rats [54,55]. Furthermore, Lin et al. (2010) found that FA (10^−5^ M) increased the expression of angiogenic proteins such as vascular endothelial growth factor (VEGF) and platelet-derived growth factor (PDGF), and upregulated the number of their major regulator hypoxic-induced factor (HIF) through adjusting the VEGF/PDGF/HIF1α pathway in human umbilical vein endothelial cells (HUVECs) [56]. These studies suggest that FA might be used for the treatment of DPN by downregulating the expression of inflammatory factors and promoting angiogenesis. 

### 3.3. The Effects of FA on Diabetic Hypertension

Diabetic hypertension (DHP), as one of the complications of DM, can cause endothelial dysfunction and vascular disorder by activating the polyol pathway [57,58,59,60,61]. In the presence of hyperglycemia, hexokinase is saturated, while excess glucose fails to be digested through the glycolytic pathway but can be metabolized through the polyol pathway, along with the activation of aldose reductase, which drives the conversion of glucose into sorbitol [58]. The accumulation of sorbitol in cells leads to the upregulation of NADH/NAD ratio in the process of excessive sorbitol oxidation to fructose, which in turn causes various metabolic imbalances including vascular dysfunction [59,62]. It has been reported that FA possesses an inhibitory effect on aldose reductase in the polyol pathway [63]. L-arginine is catalyzed by nitric oxide synthase (NOS) to produce nitric oxide (NO) in response to a variety of factors, including mechanical shear stress [64]. NO can keep endothelial cells in a natural resting state of vasodilation. It is well-known that NO can diffuse into the underlying smooth muscle from endothelial cells and stimulate guanylate cyclase to increase cyclic guanosine monophosphate(cGMP) production, which further induces the relaxation of vascular smooth muscle and causes vasodilation [64,65,66,67]. Hypertension is associated with NO deficiency, which is an important risk factor for atherosclerosis and endothelial dysfunction [68]. Hypertension-induced vascular dysfunction may relate to vascular remodeling and microvascular rarefaction caused by chronically elevated systemic arterial blood pressure [57,69,70]. Moreover, STZ is known to induce an increase in both diastolic and systolic blood pressure, and the administration of FA (20 mg/kg) by oral gavage for 6 weeks to STZ-induced diabetic rats improved endothelial-dependent relaxation, NO production and vasoconstriction capacity in isolated aorta [71]. Badawy et al. (2013) suggested that FA ameliorated diabetes-induced impairment of endothelial-dependent relaxation and promoted NO production [71]. Additionally, Yin et al. (2014) found that FA combined with astragaloside IV improved the release of NO and endothelial nitric oxide synthase (eNOS) to alleviate vascular endothelial dysfunction through the NF-κB pathway, thereby exerting inhibitory effects on hypertension [72]. 

### 3.4. The Effects of FA on Diabetic Retinopathy

Diabetic retinopathy (DR) is charactered by retinal microvascular changes, leading to a certain degree of retinopathy and visual impairment [73]. Many pathways, including the renin–angiotensin pathway, the kallikrein–kinin system and the AGEs pathway, are known to be associated with the pathogenesis of DR [74]. The Chinese medicine formula He-Ying-Qing-Re Formula (HF), with FA as a major component, is known to attenuate DR by increasing the expression of tight junction proteins zonula occluden-1 (ZO-1) and activating the Akt signaling pathway in human retinal endothelial cells (HRECs) at a concentration of 100 μg/mL. Furthermore, HF was found to attenuate retinal vascular degeneration in retinal vasculature through upregulating the level of claudin-1 and inhibiting the activation of AGEs receptors in STZ-induced diabetic C57BL/5J mice at a dose of 100 mg/kg by gavage daily for 4 weeks [75]. Importantly, FA significantly ameliorated the expression of apoptosis-related markers in retinal pigment epithelial cells, such as protein 53 (p53), B cell lymphoma 2 (Bcl2) and Bcl2-associated x (Bax) [76]. These results indicate the promising role of FA in the alleviation of DR.

### 3.5. The Effects of FA on Wound Healing with DM

In diabetic patients, the skin wound healing process is delayed under the stimulation of the hyperglycemic environment caused by complex factors including abnormal angiogenesis, and impaired function of keratinocytes and fibroblasts [77]. FA has the characteristics of improving blood fluidity, inhibiting platelet aggregation, and exhibiting strong antioxidant activity [77]. Additionally, the increased expression of angiogenesis-related VEGF and PDGF in endothelial cells might be involved in the pharmacological mechanism of FA in wound healing [56]. Moreover, FA-loaded electrospun biomimetic multifunctional nanofibers were found to accelerate wound healing in STZ-injected diabetic albino Wistar rats by exerting antibacterial activity against *Staphylococcus aureus* and *Pseudomonas aeruginosa* [78]. Thus, FA may improve diabetes-induced impaired wound healing by promoting angiogenesis, decreasing oxidative stress and inhibiting bacterial growth during wound healing.

### 3.6. The Effects of FA on Diabetic Cardiomyopathy

Cardiac diseases (including coronary heart disease and stroke) are the most common non-communicable diseases globally [79]. Diabetic patients have a greater risk of heart disease and a higher mortality. Emerging evidence suggests that people with diabetes have almost more than twice the risk of developing heart failure as compared to people without diabetes [80]. Sustained hyperglycemia aggravates reactive oxygen species (ROS) production which leads to the changes in myocardium structure and function [81]. Diabetic cardiomyopathy (DCM) is characterized by markedly increased in levels of low-density lipoprotein (LDL), glucose, glycated hemoglobin (HbA1c), and fibrotic markers insulin-like growth factor (IGF)-B7 and transforming growth factor (TGF)-β1, along with severe diastolic dysfunction [82]. With a high level of glucose in diabetic patients, the opening of the mitochondrial permeability transition pore (mPTP) and depolarization of the mitochondrial membrane potential eventually lead to increased mitochondrial ROS production [83,84]. The mitochondrial ROS can disturb the function of cardiomyocytes via inducing DNA damage, inflammation and apoptosis [85,86,87,88]. It has been demonstrated that FA can protect cardiac mitochondria in different ways, such as by inhibiting mPTP opening, disrupting mitochondrial membrane potential and reducing mitochondrial ROS production [84]. Additionally, another study suggested that oral gavage with 50 mg/kg FA for 8 weeks improved DCM via activating cardiac PI3K, Akt and glycogen synthase kinase 3β (GSK-3β), and ameliorating the translocation of glucose transporter type 4 (GLUT4) to the cardiac membrane by activating PI3K/Akt signaling pathway in STZ-induced Wistar rats [89]. Overall, based on its antioxidant and hypoglycemic effects, FA could be used as a potential therapeutic drug for DCM.

### 3.7. The Effects of FA on Diabetic Alzheimer′s Disease

Cognitive impairment has been recognized as a typical characteristic of neurodegenerative disease in DM, and previous studies have shown that patients with diabetes have an increased risk of behavioral delay and learning and memory deficits, which are closely related to neurochemical and neurostructural changes, and this cognitive dysfunction may be a risk factor that causes Alzheimer′s disease [90,91]. Long-term chronic hyperglycemia promotes changes in inflammatory response and Alzheimer′s disease-like neurodegenerative changes, and impairs insulin signaling pathways, leading to cognitive dysfunction in diabetic rats. A study revealed that the oral administration of FA at a dosage of 15 and 30 mg/kg for 4 consecutive weeks could exhibit beneficial effects on diabetes-induced cognition lesions in STZ and high glucose-fat diet-treated SD rats, which was due to its involvement in regulating the accumulation of protein tyrosine phosphatase 1B (PTP1B) and amyloid-β (Aβ) accumulation, as well as blocking the neuroinflammation and activating insulin signaling pathway [9]. 

In addition, we summarize papers in detail that verified the major effects of FA in management of diabetes complications in Table 2.

## 4. Summary and Outlook

**Figure 4:**
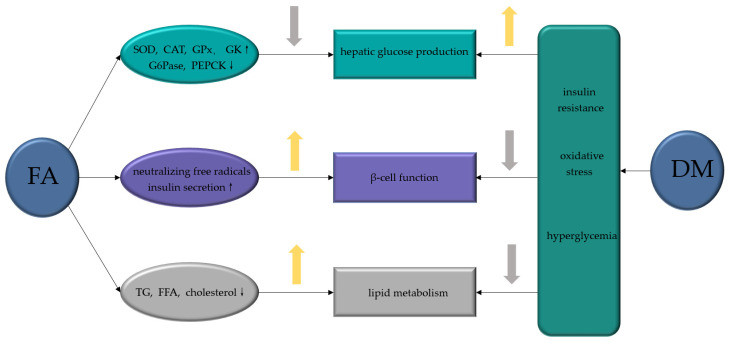
The mechanisms of FA in alleviating hepatic glucose production and improving β-cell function and lipid metabolism during the development of DM. FA, ferulic acid; DM, diabetes mellitus; SOD, superoxide dismutase; CAT, catalase; GPx, glutathione peroxidase; GK, glucokinase; G6Pase, glucose-6-phosphatase; PEPCK, phosphoenolpyruvate carboxykinase; TG, triglycerides; FFA, free fatty acid.

**Figure 5:**
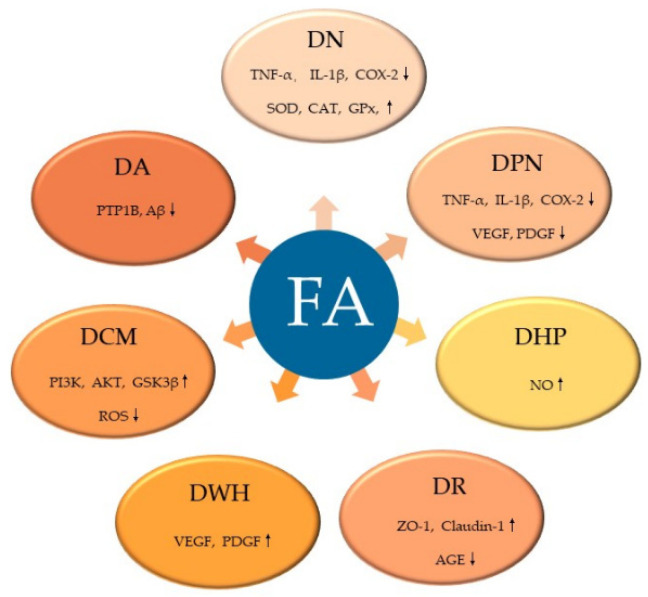
FA ameliorates diabetic complications via different molecular targets. FA, ferulic acid; SOD, superoxide dismutase; CAT, catalase; GPx, glutathione peroxidase; TNF-α, tumor necrosis factor-α; IL-1β, interleukin-1β; COX-2, cyclooxygenase-2; VEGF, vascular endothelial growth factor; PDGF, platelet derived growth factor; NO, nitric oxide; AGE, advanced glycation end products; ZO-1, zonula occluden-1 (ZO-1); PI3K, phosphoinositide-3 kinase; AKT, protein kinase B; ROS, reactive oxygen species.

FA, a common natural product with few toxic and side effects, exerts hypoglycemic effects by attenuating the dysfunction of various target cells in the treatment of DM (Figure 4). FA can ameliorate hepatic glucose prod disorder caused by insulin resistance through increasing GK, GPx, SOD, and CAT and decreasing the activities of G6Pase and PEPCK. By neutralizing free radicals, reducing lipid peroxidation and promoting insulin secretion, FA reduces the β-cell dysfunction caused by diabetes and the excessive production of free radicals, which is also related with its inhibition of the excess production of TG or FFA caused by diabetes. Moreover, FA exerts a variety of effects on the complications of DM (Figure 5). FA ameliorates DN by increasing the activity of SOD, CAT and GPx, attenuates DPN by alleviating inflammatory response, mitigates DHP by promoting NO production, controls DR by inhibiting the activation of AGEs receptors and the Akt signaling pathway, promotes angiogenesis during wound healing, and treats DCM by inhibiting ROS production. Among the signaling pathways regulated by FA administration, the NF-κB signaling pathway is involved in the pathology of different diabetes complications, such as DN, DPN, and DHP, and it also plays a role in β-cell destruction by regulating the inflammatory response of β-cells. Similarly, the PI3K/Akt pathway is known to be a major effector of insulin metabolism, and has a leading effect in the development of DN and DCM. Therefore, both the NF-κB and PI3K/Akt pathways might be important mechanisms of action of FA in the management of DM and its complications.

Overall, this review offers a summarized overview on the therapeutic effects of FA on DM and its complications, which can contribute to the understanding of the biological activity of FA. Although a variety of studies have been reported confirming the efficacy of FA in the management of diabetes and its complications, the clinical effects of FA still need to be tested in the future. 

## Figures and Tables

**Table 1:** Major effects of FA on diabetes control.

Diabetic Models	Dosage Range	Major Reported Antidiabetic Endpoints	References
STZ (40 mg/kg i.p. injection) in Wistar rats	10 mg/kg for 45 days, i.v.	Increasing the activities of antioxidant enzymes such as GPx, SOD and CAT, neutralizing STZ-induced free radicals in the pancreas.	[20]
High-fat diet + fructose in Wistar rats	50 mg/kg for 30 days, i.g.	Decreasing hepatic glucose production in the liver tissue, returning blood glucose, serum insulin, glucose tolerance and insulin tolerance to the normal range.	[11]
High-fat diet in male C57BL/6N mice	0.5% supplemented diet ad libitum for 7 weeks	Increasing the activity of hepatic GK enzyme, and reducing the activities of G6Pase and PEPCK.	[19]
High-fat diet in gestational SD rat	20 mg/kg for 12 weeks, i.g.	Inhibiting the apoptosis of β-cells in pancreatic islets.	[28]
STZ (60 mg/kg i.p. injection) in Wistar rats	50 mg/kg for 8 weeks, i.g.	Reducing the lipid peroxidation in pancreatic tissues.	[29]
STZ (60 mg/kg i.p. injection) in Wistar rats	10 mg/kg for 3 weeks (in combination with metformin), i.g.	Improving impaired β-cell regeneration.	[30]
High-fat diet in ICR mice	25 and 50 mg/kg for 8 weeks, i.v.	Reducing the levels of plasma TG, FFA, cholesterol and phospholipids, decreasing expression of SREBP1c, FAS, ACC, CPT1a, and PPARα.	[35]
STZ (40 mg/kg i.p. injection) in Wistar rats	10 mg/kg for 45 days, i.g.	Reducing the levels of TBARS, hydroperoxides and FFA in the liver.	[37]
STZ (150 mg/kg i.p. injection) in ICR mice	0.01% supplemented diet ad libitum for 7 weeks	Reducing TBARS in brown adipose tissue.	[38]

**Table 2:** Major effects of FA in management of diabetes complications.

Diabetic Models (In Vitro and In Vivo)	Dosage Range	Diabetic Complications	Beneficial Effects and Involved Mechanisms	References
STZ (50 mg/kg i.v. injection) in male SD rats	100 mg/kg for 8 weeks, i.g.	Diabetic nephropathy	Improving the renal organ coefficient, increasing activities of SOD, CAT, and GPx.	[39]
STZ (50 mg/kg i.p. injection) in Wistar rats	50 mg/kg for 8 weeks, i.g.	Diabetic nephropathy	Ameliorating renal cell apoptosis, inflammation and defective autophagy, modulating advanced AGEs, NF-κB, MAPKs, P38, JNK Erk1/2 signaling pathways.	[43]
Sucrose (30% in drinking water) in OLETF rats	10 mg/kg for 20 weeks, i.g.	Diabetic nephropathy	Reducing oxidative stress, inflammatory response, and decreasing the ACR, urinary MDA and MCP-1 levels.	[48]
STZ (55 mg/kg i.p. injection) in SD rats	100 mg/kg for 4 weeks (in combination with insulin), i.g.	Diabetic neuropathy	Downregulating the levels of TNF-α and IL-1β, decreasing COX-2 activity in the sciatic nerve.	[54]
STZ (50 mg/kg i.p. injection) in male Wistar rats	20 mg/kg for 6 weeks, i.g.	Diabetic hypertension	Improving endothelial-dependent relaxation, NO production and vasoconstriction capacity in isolated aorta.	[71]
STZ (55 mg/kg i.p. injection) in C57BL/5J mice	100 mg/kg HF containing FA as a major component for 4 weeks, i.g.	Diabetic retinopathy	Attenuating retinal vascular degeneration through upregulating the level of claudin-1 and inhibiting the activation of AGEs receptors	[78]
HG (30 mmol/L) induced ARPE-19 cells	10 mmol/L	Diabetic retinopathy	Ameliorating the expression p53, Bcl2 and Bax.	[76]
STZ (50 mg/kg i.p. injection) in Wistar rats	10 and 20 mg/kg for 14 days, i.g.	Diabetic wound healing	Improving blood fluidity, inhibiting platelet aggregation, and exhibiting strong antioxidant activity.	[77]
STZ (50 mg/kg i.p. injection) in Wistar rats	50 mg/kg for 8 weeks, i.g.	Diabetic cardiomyopathy	Activating cardiac PI3K, Akt and GSK-3β, and ameliorating the translocation GLUT4.	[89]
STZ (35 mg/kg i.p. injection) and high-glucose-fat diet in Wistar rats	15 and 30 mg/kg for 4 weeks, i.g.	Diabetic Alzheimer′s disease	Regulating the accumulation of PTP1B and Aβ, as well as blocking neuroinflammation and activating the insulin signaling pathway.	[9]

## Data Availability

The original data used in this paper are all from published papers and can be obtained from references in the text.
